# AntiPD-L1 antibody conjugated Au-SPIOs nanoplatform for enhancing radiosensitivity and triggering anti-tumor immune response

**DOI:** 10.1038/s41598-022-23434-z

**Published:** 2022-11-15

**Authors:** Chengrun Du, Jianyun Jiang, Caifeng Wan, Guangsen Pan, Fangfang Kong, Ruiping Zhai, Chaosu Hu, Hongmei Ying

**Affiliations:** 1 Department of Radiation Oncology, Fudan University Shanghai Cancer Center, Shanghai, 200032 China; 2 Department of Oncology, Shanghai Medical College, Fudan University, Shanghai, 200032 China; 3 Shanghai Key Laboratory of Radiation Oncology, Shanghai Clinical Research Center for Radiation Oncology, Shanghai, 200032 China; 4 Department of Ultrasound, School of Medicine, Renji Hospital, Shanghai Jiaotong University, Shanghai, China

**Keywords:** Cancer therapy, Tumour immunology, Cancer, Oncology

## Abstract

To improve radiotherapy effect by inducing more toxicity for tumors and less for normal tissue and switching immunosuppressive microenvironment caused by expression of PD-L1 and tumor-associated macrophages (TAMs) to immunoreactive microenvironment, we designed a PD-L1-targeted nanoplatform consisting of gold nanoparticles and superparamagnetic iron oxide nanoparticles (antiPD-L1-SPIOs@PLGA@Au). In vivo T2-weighted images, the best contrast effect of tumor was achieved two hours after intravenous injection of antiPD-L1-SPIOs@PLGA@Au. The tumor control caused by irradiation combined with antiPD-L1-SPIOs@PLGA@Au was better than that by radiotherapy alone in clone formation assay and B16F10 subcutaneous tumor model. Radiosensitivity enhancement induced by the addition of antiPD-L1-SPIOs@PLGA@Au was achieved by increasing ROS production and attenuating DNA damage repair. AntiPD-L1-SPIOs@PLGA@Au could promote the polarization of tumor-associated macrophages (TAMs) to M1 and reverse the immunosuppression caused by TAMs. By increasing the expression of CRT in tumor and blocking the PD-L1/PD pathway, antiPD-L1-SPIOs@PLGA@Au with radiation activated the anti-tumor immune response. In conclusion, antiPD-L1-SPIOs@PLGA@Au could be used as a radiosensitizer and a MRI contrast targeting PD-L1, with the functions of blocking the PD-L1/PD-1 immune checkpoint pathway and reversing the immunosuppression caused by TAMs.

## Introduction

Radiotherapy (RT) is a mainstream cancer treatment strategy that has been extensively used in clinic to treat 65–75% of local solid tumors with curative or palliative intent^1^. The goal of radiotherapy is to maximize the radiation dose to the tumor volume while limiting off-target side effects. With the application of more precise technology, such as intensity-modulated radiation therapy (IMRT) and stereotactic body radiation therapy (SBRT), RT has brought more benefit for cancer patients. However, certain cancer patients still faced treatment failure after radiotherapy, especially those patients with malignant tumor possessing the nature of radiation resistance. With the advantage of greater absorption and deposition of energy in surrounding tissues, gold nanoparticles (AuNPs) have been most extensively studied as radiosensitizer^2,3^. Superparamagnetic iron oxide nanoparticles (SPIOs) are capable of generating T_2_-weighted contrast enhancement in magnetic resonance imaging (MRI). A nanoplatform consisting of AuNPs and SPIOs can augment cancer treatment by facilitating imaging and increasing the efficacy of therapy, ultimately possessing radiotheranostic property^4^. Moreover, SPIO recently was found to be able to polarize tumor-associated macrophages (TAMs) from M2-like type (protumor) to M1-like type (antitumor), reversing immunosuppressive microenvironment, which is crucial to the effect of radiotherapy^5^.

Programmed death-ligand 1(PD-L1), a type I transmembrane protein, is expressed in cytoplasm and cell surface of tumor^6,7^. Inhibition of PD-1/PD-L1 axis has shown to augment cytotoxic T cell responses in various types of tumors^8^. Radiation induces a local inflammatory response that could enhance the infiltration of tumor-specific T cells. However, it also simultaneously induces PD-L1expression in the tumor microenvironment that markedly weakens radiation-induced antitumor immunity^9^.

In this work, to achieve enhancing radiosensitivity and triggering antitumor immune response simultaneously, we fabricated multifunctional poly (lactic-co-glycolic acid) (PLGA) nanoparticles using the single emulsion oil-in- water (O/W) solvent evaporation method. AuNPs were coated on the surface of PLGA, whereas SPIOs were encapsulated inside the core. The nanoparticle was conjugated with antiPD-L1 antibody to inhibit the PD-1/PD-L1 axis and increase the target specificity. The abilities of antiPD-L1-SPIOs@PLGA@Au to target PD-L1 and radiosensitize B16F10 tumor in vitro/vivo were evaluated and the underlying mechanisms were explored.

## Materials and methods

### Materials

Oleic acid-coated superparamagnetic iron oxide nanoparticles (SPIOs, 7–10 nm) were purchased from So-Fe Biomedical Co., Ltd. (Shanghai, China). Tetrachloroauric (III) acid trihydrate (HAuCl4·3H2O), poly (lactic-co-glycolic acid) (PLGA) and polyallylamine hydrochloride (PAH) were purchased from Sigma-Aldrich Trading Co., Ltd. (Shanghai, China). Polyvinyl alcohol (PVA), 1-(3-dime-thylaminopropyl)-3-ethylcarbodiimide hydrochloride (EDC), and N-hydroxysuccinimide (NHS) were obtained from Aladdi Chemistry Co., Ltd. (Shanghai, China). All other reagents used were of analytical grade. The information about the antibodies used was provided in supplementary materials.

### Synthesis and characterizations of antiPD-L1-SPIOs@PLGA@Au

AntiPD-L1-SPIOs@PLGA@Au was fabricated in three steps: preparation of SPIOs@PLGA, formation of SPIOs@PLGA@Au and conjugation of antiPD-L1 antibody. The details were based on previous reports^10^. Briefly, SPIOs@PLGA was obtained with the mixture of oleic-acid-coated SPIOs (10 mg) and 100 mg PLGA using single emulsion oil-in-water solvent evaporation method. And then Au nanoshell was coated on the surface of SPIOs@PLGA by reducing HAuCl_4._ Finally, coupling antiPD-L1 antibody with SPIOs@PLGA@Au was achieved by adding SH–poly (ethylene glycol) (PEG)–carboxylic acid (COOH) (SH–PEG–COOH) (5 mg) according to carbon diimide method.

Field emission scanning electron microscopy (FESEM, Hitachi S-4800) and transmission electron microscope (TEM, JEM-2100) were used to evaluate the morphology and internal structure antiPD-L1-SPIOs@PLGA@Au. The attached energy-dispersive X-ray spectroscopy (EDS) was used to evaluate the corresponding elements. The size distribution and zeta potential of antiPD-L1-SPIOs@PLGA@Au were characterized using dynamic laser scattering (DLS) instrument (Zetasizer Nano ZS3690).

### Cell culture

B16F10 and HUVECs (Human Umbilical Vein Endothelial Cells) cell line were purchased from the Institute of Biochemistry and Cell Biology (Shanghai, China) and were cultured at 37 °C in DMEM (Clonogenic survival Gibco Life Technologies, Grand Island, NY, USA) containing 10% fetal bovine serum (FBS) and 1% penicillin–streptomycin at 37 °C in a humidified incubator containing 5% CO_2_.

### Murine tumor model

All procedures were performed with approval from Laboratory Animal Resources Division of Fudan University and in accordance with relevant regulations and guidelines, including the ARRIVE guidelines. C57BL/6 mice (Female, 6–8 weeks old,) were inoculated with B16F10 cells (2 × 10^5^ B16F10) subcutaneously at the right flank. Tumors were allowed to grow until their volume was approximately 100 mm^3^. Tumor volume was calculated using the formula: major axis × (minor axis)^2^ × 0.5. Mice were killed when tumors volume reached 3000 mm^3^ or if the tumors ulcerated. Randomization was based on numbers generated by the standard = RAND() function in Microsoft Excel.

### In vitro cytotoxicity assessment

To evaluate the in vitro cytotoxicity, B16F10 cells and HUVECs at a density of 1 × 10^4^/well in 96-well plates were incubated with antiPD-L1-SPIOs@PLGA@Au with different concentrations (0, 10, 20, 50, 100, and 200 μg/ml). Cell counting kit-8(CCK8) proliferation assays (Dojindo Molecular Technologies Inc., Japan) were performed at 12 h and 24 h after incubation.

### In vivo toxicity study

C57BL/6 mice were injected with antiPD-L1-SPIOs@PLGA@Au (200 μl, 7 mg/ml). Toxicities of antiPD-L1-SPIOs@PLGA@Au in mice were evaluated 7 days post injection. Blood samples collected from the ophthalmic vein under anesthesia were used to test blood biochemistry indexes. Major organs including heart, liver, spleen, lung, and kidney were stained with hematoxylin and eosin (H&E). Mice treated with saline were used as control group.

### Targeting specificity in vitro

B16F10 cells were seeded in confocal cell-culture dishes at a density of 2 × 10^4^ cells/well. FITC-labelled antiPD-L1-SPIOs@PLGA@Au was added and incubated for 4 h. In the antibody blocking group, 5 μl antiPD-L1 antibody without FITC was used to block PD-L1 before adding FITC-labelled antiPD-L1-SPIOs@PLGA@Au. After washed three times with PBS, the cells were fixed with 4% paraformaldehyde for 15 min. Cell nuclei was stained with nucleus staining agent DAPI for 10 min and observed under a confocal laser scanning microscopy (CLSM).

### Magnetic resonance imaging

T_2_ MR imagings with different Fe concentrations (0, 0.002, 0.004, 0.006, and 0.008 mM) were conducted with a 0.5 T MRI scanner (Niumag MiniMR-60). T_2_ relaxivity was measured by linearly fitting the reverse T2 relaxation times as functions of the Fe concentration.

In vivo imaging, B16F10-bearing mice were classified into three groups. The first group was injected with antiPD-L1-SPIOs@PLGA@Au (200 μl, 7 mg/ml), the second group with SPIOs@PLGA@Au and the third group with saline. Tumors were imaged before and at various time points (0.5 h, 1 h, 2 h, 4 h, 6 h) after injection (200 μl, 7 mg/ml) using a Bruker 7.0 T MRI scanner (BIOSPEC70/20USR).

### Radiosensitization of melanoma cells with antiPD-L1-SPIOs@PLGA@Au.

B16F10 cells were incubated with SPIOs@PLGA@Au and antiPD-L1-SPIOs@PLGA@Au for 24 h. Cells were irradiated at different doses (2 Gy, 4 Gy, 6 Gy, 8 Gy) with 220 kV X-ray at a dose rate of 2 Gy/min using the Small Animal Radiation Research Platform (SARRP) at the Fudan University Shanghai Cancer Center. After irradiation, cells were trypsinized and counted. Known numbers were then replated and returned to the incubator to allow macroscopic colony development. Colonies were counted after 7 days, and the plating efficiency and surviving fraction for given treatments were calculated. The mean lethal dose (Do), survival fraction (SF) and SF2 sensitized ratio of B16F10 cells in different treatment groups were calculated by fitting the cell survival curve with multi target single hit equation: SF = 1 − (1 − exp(D /D0))^N.

### Therapeutic efficacy and survival study in B16F10-tumor-bearing mice

Mice were anesthetized intraperitoneally with 1 ml/kg of a solution containing 13 mg of ketamine and 86 mg of xylazine per ml. Mice were randomly divided into four groups composed of 4 mice each: saline, RT alone, RT plus SPIOs@PLGA@Au and RT plus antiPD-L1-SPIOs@PLGA@Au. Animals treated with SPIOs@PLGA@Au and antiPD-L1-SPIOs@PLGA@Au (200 μL, 7 mg/ml, IV injection) were exposed to radiation at the time point determined by MRI. The total dose of 15 Gy was given in 1 fraction. After the treatments, the tumor size and body weight were recorded every 2 days.

### ROS measurement in vitro

Cells were seeded in triplicate in 12-well plates 24 h prior to treatment, pretreated with SPIOs@PLGA@Au or antiPD-L1-SPIOs@PLGA@Au for 24 h, and then irradiated. After irradiation, fresh medium containing 4 μM CM-H2DCFDA (ThermoFisher, C6827) for ROS measurement was added to each well. After incubation for 30 min in a humidified incubator (at 37 °C, 5% CO2), the cells were washed with PBS and trypsinized to obtain a cell suspension. ROS was analyzed by Bio-Rad microplate reader (Biotek Synergy 4) at 488 nm or visualized by CLSM.

### γ-H2AX formation experiment

B16F10 cells were cultured in a confocal culture dish with inoculation density of 5 × 10^4^. After 24 h of culture, the cells were divided into 3 groups: radiation alone, SPIOs@PLGA@Au (200 μg/ ml) with radiation and antiPD-L1-SPIOs@PLGA@Au (200 μg/ ml) with radiation, with 3 replicated holes in each group. The cells were irradiated with 220 kV X-ray at a single dose of 6 Gy. Twenty-four hours after irradiation, paraformaldehyde fixative was added and fixed for 10–15 min. Anti- phosphorylated histone γ-H2AX monoclonal antibody was incubated overnight in a wet box at 4 °C. Diluted CY3-labeled sheep anti-mouse secondary antibody (1:1000) was used as second antibody. The number of fluorescence bright spots in at least 50 cells in each field was counted under the microscope.

### In vitro evaluation of the polarization of macrophages

Macrophages were obtained by stimulating mouse bone marrow mesenchymal stem cells with M-CSF. A transwells two-compartment petri dish system with an interseptal aperture of 0.4 micron was used. In this dish, molecules can pass freely through membrane pores, but cells cannot. Mouse macrophages were cultured in the lower chamber and upper chamber were divided into 4 groups: PBS group, tumor cell B16F10 group, tumor cell B16F10 + SPIOs@PLGA@Au mixed solution group and tumor cell B16F10 + antiPD-L1-SPIOs@PLGA@Au mixed solution group. After 24 h co-culture, macrophages in the lower chamber were isolated and the difference in the number of CD11^+^F4/80^+^CD86^+^ (M1 type) and CD11^+^F4/80^+^CD206^+^ (M2 type) macrophages were analyzed by flow cytometry.

### Immunofluorescence assay

To characterize the TAMs polarization markers (F4/80, CD 86 and CD 206), the infiltration of CD4^+^ and CD8^+^ T cells, the expression of CRT in tumor section, immunofluorescence assays were performed. Frozen tissue sections of 6 mm thickness were prepared, air-dried for at least 1 h and then fixed in acetone for 10 min at − 20 °C. After blocking with 20% donkey serum, the sections were incubated with primary antibodies overnight at 4 °C, followed by incubation with dye-conjugated secondary antibodies for 1 h. After staining with DAPI for another 10 min, the sections were then washed twice with PBS and observed under CLSM (Olympus, IX83).

### Flow cytometry

Cells were stained with the following fluorochrome-conjugated antibodies: CD3, CD4, CD8, CD11 B, F4/80, CD86 and CD206, and then tested by flow cytometry. Data analysis was carried out using FlowJo software.

### Enzyme-linked immunosorbent assay (ELISA)

The sera of mice in each treatment group were collected from the ophthalmic vein 7 days after treatments and assayed for mouse interferon-γ (IFN-γ), tumor necrosis factor α (TNF-α), and interleukin 12 (IL-12) levels using quantitative enzyme-linked immunosorbent assay kit (R&D systems), following validation of each ELISA according to the manufacturer's instructions. Absorbance was read using a Bio-Rad microplate reader (Biotek Synergy 4) at 488 nm.

### Statistical analysis

All in vitro experiments were carried out in triplicate. Data were expressed as the mean ± standard error (SE). Comparison between two groups was performed using an unpaired two-tailed t-test. When comparing multiple groups, a one-way analysis of variance (ANOVA) was used. Kaplan–Meier survival curves were analyzed using the log-rank test for the survival data. Differences were considered statistically significant when *P* < 0.05.

## Results

### Characterizations of antiPD-L1-SPIOs@PLGA@Au

**Figure 1:**
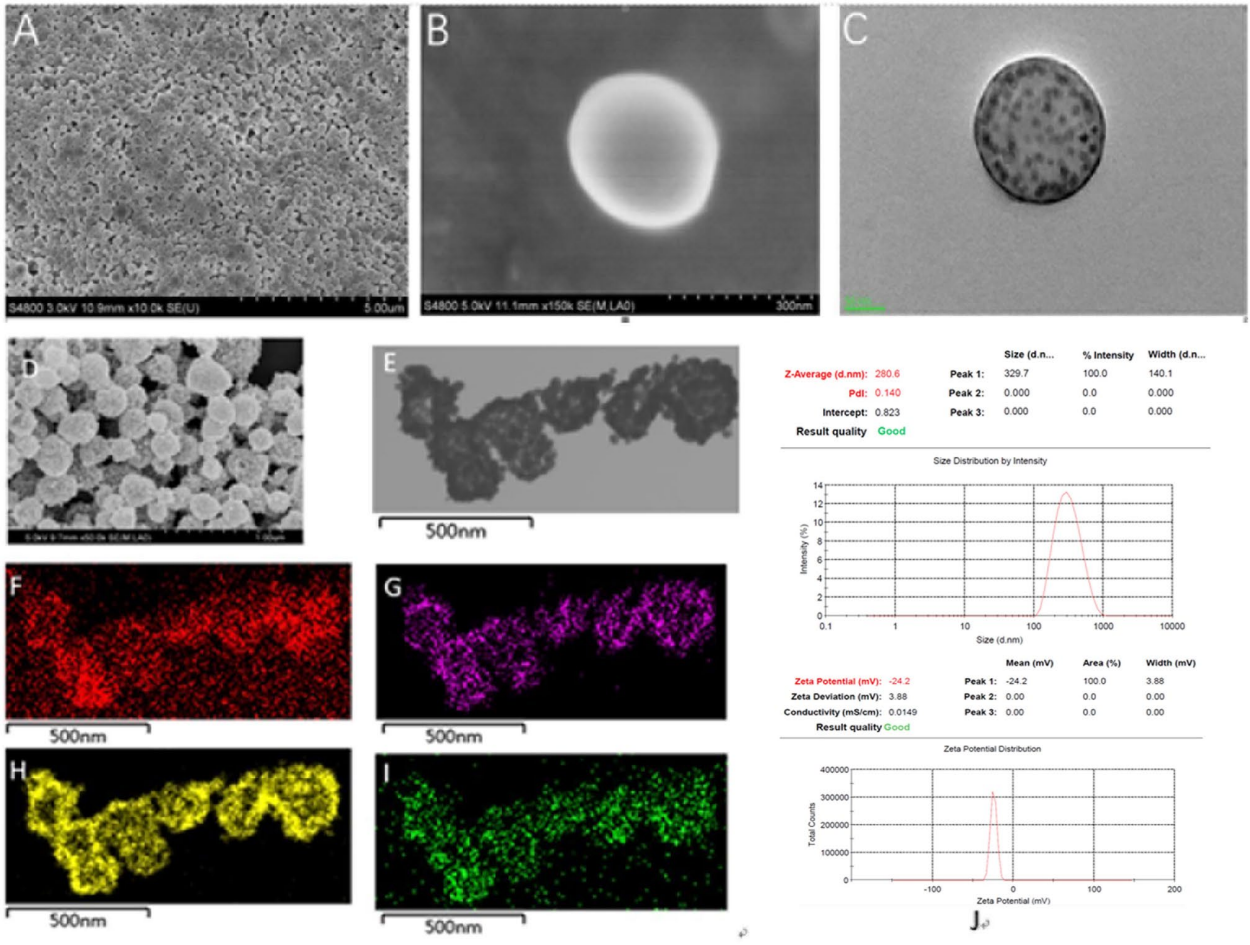
(**A**) SEM image of SPIOs@PLGA (scale bar = 5 μm); (**B**) SEM image of SPIOs@PLGA (scale bar = 300 nm); (**C**) TEM image of SPIOs@PLGA; (**D**) SEM image of SPIOs@PLGA@Au (scale bar = 1 μm); (**E**) TEM image of SPIOs@PLGA@Au; (**F**–**I**) EDS element mapping images of SPIOs@PLGA@Au exhibiting the presence of characteristic C, Fe, Au and O elements(**F**:C; **G**:Fe; **H**:Au; **I**:O) ; (**J**) Average size and size distribution of antiPD-L1-SPIOs@PLGA@Au.

SPIOs@PLGA exhibited well-defined spherical shape and homogenous size (Fig. 1A–C). TEM images illustrated the deep gray spots in the shell and core region of the nanoparticle. After Au NPs were coated on the surface of SPIOs@PLGA, the regular spherical morphology was well maintained but the surface roughness increased due to deposition of aggregated Au NPs in SEM images (Fig. 1D). As TEM images (Fig. 1E) illustrated, dense Au nanoparticles were distributed homogeneously. EDS elements mappings (Fig. 1F–I) of SPIOs@PLGA@Au clearly revealed the presence of characteristic C, Fe, Au and O elements (F:C; G:Fe; H:Au; I:O) .Under confocal laser scanning microscopy, it was observed that the FITC-labeled antibody of antiPD-L1-SPIOS@PLGA@Au was successfully connected (Fig. S1).

Average size of antiPD-L1-SPIOS@PLGA@Au NCs was 280.6 nm with a polydispersity index of 0.14 (Fig. 1J), and zeta potential was about − 24.2 mV. Notably, antiPD-L1-SPIOs@PLGA@Au was highly stable in storage (PBS, 4 °C) as evidenced by the unchanged diameter for 7 days (Fig. S2). Radiation of 6 Gy could cause the rupture of Au shell. The Au shell was break into smaller Au debris and SPIOs would be released (Fig. S2).

### The cytotoxicity of antiPD-L1-SPIOs@PLGA@Au

As shown in Fig. S3, the cell viability of B16F10 cells and HUVECs remained more than 85% after incubation with antiPD-L1-SPIOs@PLGA@Au, even at the highest concentration (200 μg/ml) for 24 h.

All measured parameters of mice were normal, indicating that antiPD-L1-SPIOs@PLGA@Au would not induce significant systemic side effects to mice (Fig. S4). The histopathology of the heart, lung, liver, spleen and kidney was analyzed to assess the biosafety of nanoparticles. The nanoparticles showed no substantial damage to the major organs, as evidenced by the stable body weights (Fig. S4) and lack of histopathological changes in major organs (Fig. S4). The above results illustrated the good biocompatibility of antiPD-L1-SPIOs@PLGA@Au, which is an essential pre-requisite for imaging and radiosensitizing applications.

### Targeting of antiPD-L1-SPIOs@PLGA@Au to B16F10 cells

It’s demonstrated that after 4 h of incubation, antiPD-L1-SPIOs@PLGA@Au could be detected on the cell membrane, where PD-L1 expressed in B16F10 cells. As shown in the bright field images, antiPD-L1-SPIOs@PLGA@Au aggregated in the way of ink spots, was mainly distributed in the cell membrane and cytoplasm. In the images of FITC pathway, green fluorescence signal was observed on the ink spots (Fig. S5).

Through blocking with antiPD-L1 antibody unlabeled with FITC, the connection between nanomaterials and B16F10 cells was significantly reduced after rinsing with PBS. No obvious ink spot and fluorescent-labeled antibody was observed on the bright field image and FITC channel (Fig. S5).

### MR imaging in vitro/vivo

**Figure 2:**
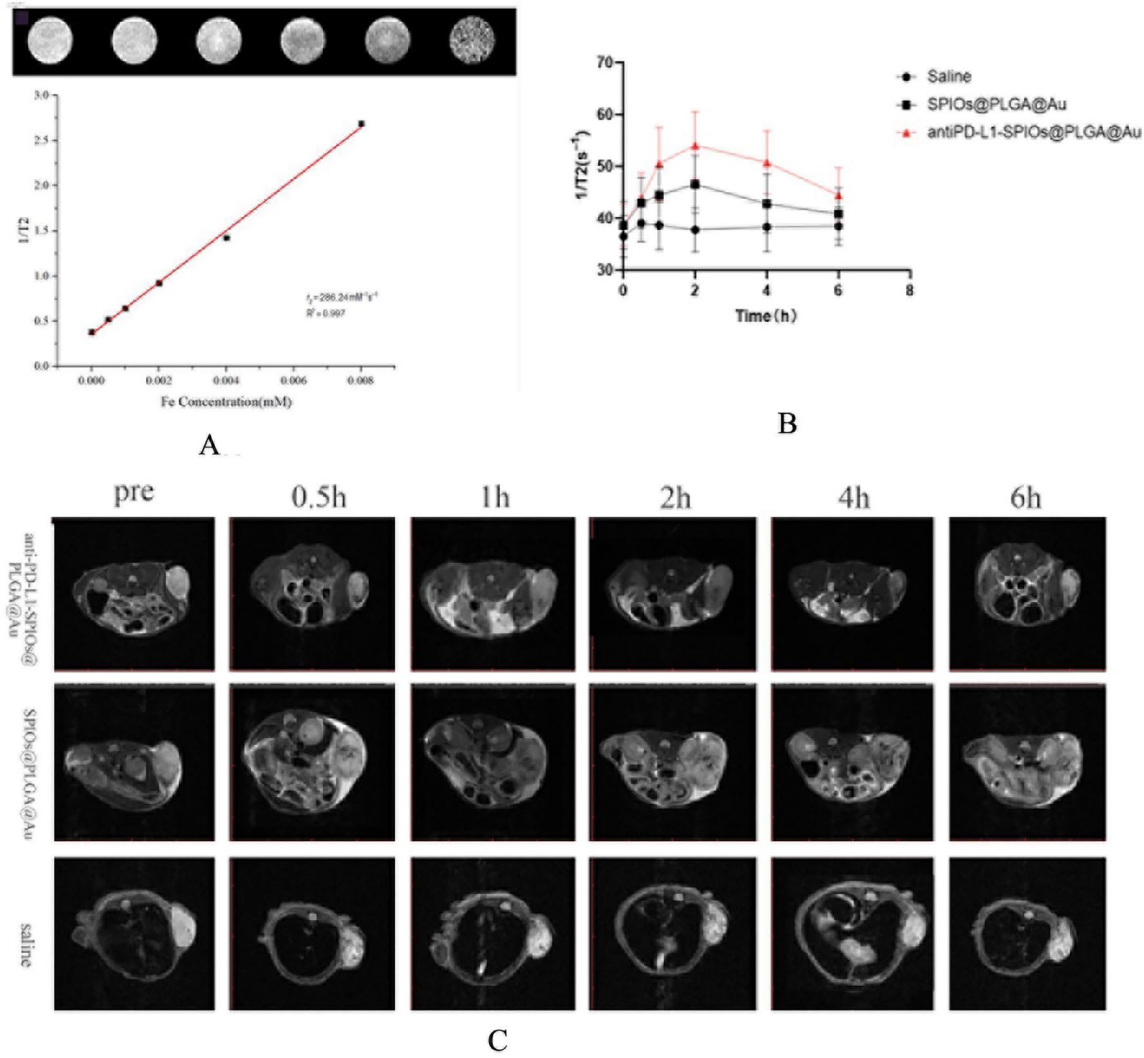
(**A**) T_2_-weighted MR images of antiPD-L1-SPIOs@PLGA@Au with increasing Fe concentrations; (**B**) T_2_ relaxation rate (1/T_2_(s-1)) corresponding to region of interest of tumor at different time points (0 h, 0.5 h, 1 h, 2 h, 4 h, 6 h) with different injections. (**C**) T_2_-weighted MR images of a B16F10 mice xenograft tumor at different time points.

Transverse relaxation rate (1/T_2_) of antiPD-L1-SPIOs@PLGA@Au aqueous solution as a function of iron concentration is shown in Fig. 2A. According to the fitting curve, T_2_ relaxivity (r2) of antiPD-L1-SPIOs@PLGA@Au aqueous solution was calculated to be 286.24 mM^−1^ s^−1^. In vivo imaging, nanoparticles contrast showed gradual enrichment and then regressed in the tumor site. The best contrast effect in tumor was at the second hour after intravenous injection of antiPD-L1-SPIOs@PLGA@Au and SPIOs@PLGA@Au (Fig. 2B,C). However, tumors in antiPD-L1-SPIOs@PLGA@Au group were still hypointense on MRI until 6 h, with higher 1/T_2_ value than that in SPIOs@PLGA@Au group, reflecting the ability to target PD-L1. In contrast, there was no significant change of signal in tumor in saline group.

### AntiPD-L1-SPIOs@PLGA@Au enhancing radiosensitivity of B16F10 melanoma

#### In vitro

Compared with radiation alone, SPIOs@PLGA@Au or antiPD-L1-SPIOs@PLGA@Au combined with radiation significantly reduced the rate of clone formation of B16F10 cells (Fig. S6) in terms of the number of colony formation and the fitted survival curve. The mean lethal doses (Do) of radiation alone, SPIOs@PLGA@Au with radiation group and antiPD-L1-SPIOs@PLGA@Au with radiation group were 1.93 Gy, 1.51 Gy and 1.34 Gy, respectively. The Do sensitization ratios of SPIOs@PLGA@Au and antiPD-L1-SPIOs@PLGA@Au groups were 1.5 and 1.34, respectively, while the SF2 sensitization ratios were 1.26 and 1.4.

#### In vivo

**Figure 3:**
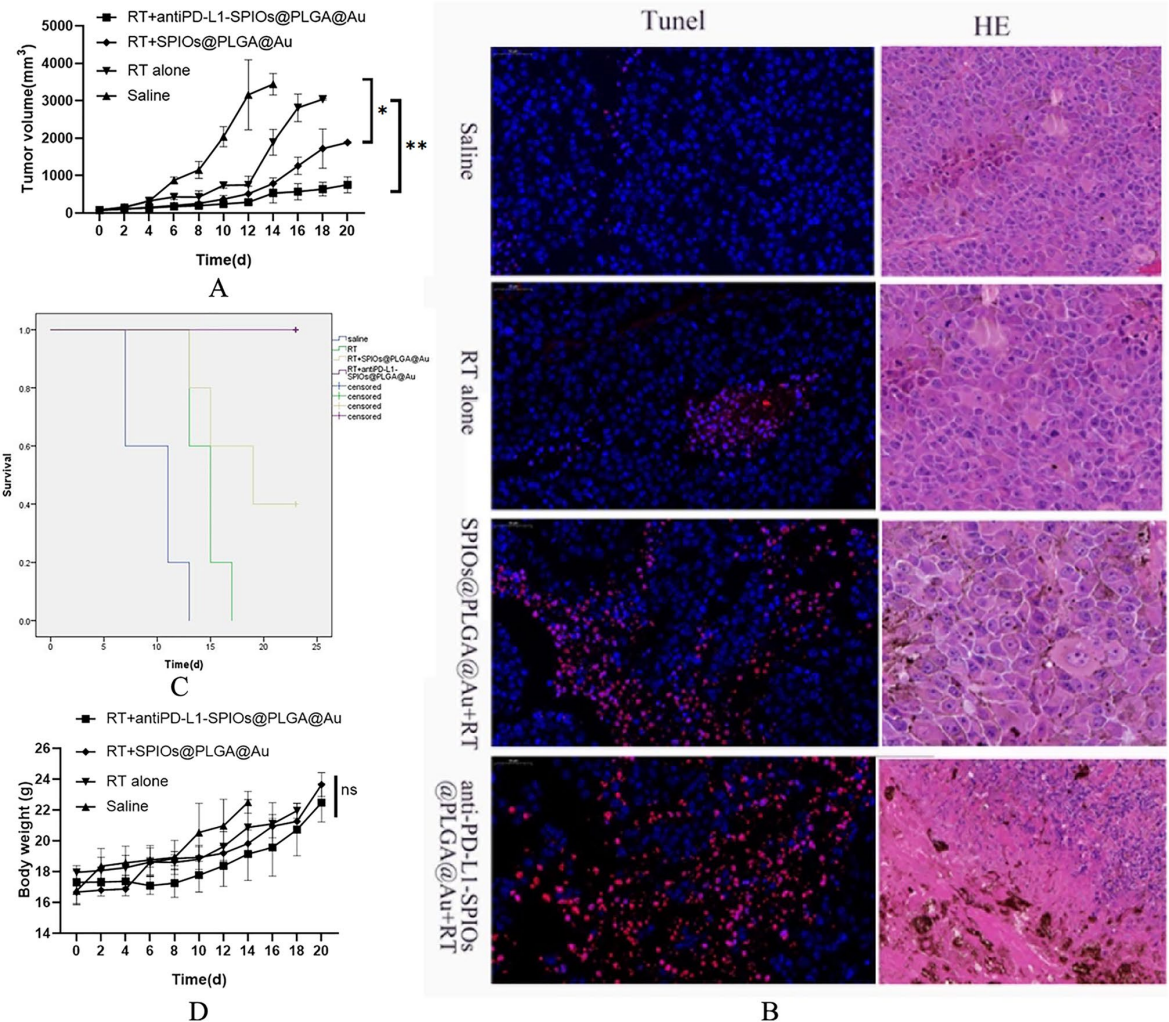
(**A**) Tumor growth in different groups (n = 4 per group) (**B**) Tunel and HE staining of tumor tissues. (**C**) Survival curves of tumor-bearing mice in different groups. (**D**) Body weight changes of B16F10 tumor-bearing mice in different groups. (ns: not significant, *p < 0.05, **p < 0.001).

Based on the results of in vivo imaging monitoring, irradiation was given at 2 h after intravenous injection. Compared with the saline group, the tumor growth in the mice given with radiation alone temporarily slowed down, but accelerated 8 days after radiation. The tumor inhibition effects of radiation combined with SPIOs@PLGA@Au or antiPD-L1-SPIOs@PLGA@Au were higher than that of radiation alone. Within 12 days after irradiation, tumor inhibition was similar between SPIOs@PLGA@Au group and antiPD-L1-SPIOs@PLGA@Au group, but 12 days after irradiation, tumor growth accelerated in SPIOs@PLGA@Au group. However, the tumor inhibition effect of antiPD-L1-SPIOs@PLGA@Au with radiation lasted until 20 days, and no obvious acceleration of tumor growth was observed (Fig. 3A). TUNEL staining showed more apoptosis in the antiPD-L1-SPIOs@PLGA@Au group. HE staining sections revealed that there were large necrotic foci of tumor tissue in the antiPD-L1-SPIOs@PLGA@Au group (Fig. 3B).During the entire observation period, only the mice in the antiPD-L1-SPIOs@PLGA@Au group did not die. The Kaplan–Meier survival curves of the different groups are illustrated in Fig. 3C. During the whole observation period, there was no significant difference in body weight among all groups (Fig. 3D).

### The mechanisms for antiPD-L1-SPIOS@PLGA@Au enhancing treatment effect

#### Reactive oxygen species (ROS)

It was found in DCFH-DA assay that more ROS was observed when either antiPD-L1-SPIOs@PLGA@Au or SPIOs@PLGA@Au was applied, compared with radiation alone (Fig. S7A). The amount of ROS produced by antiPD-L1-SPIOs@PLGA@Au with radiation was the highest among the groups (Fig. S7B).

#### DNA repair

**Figure 4:**
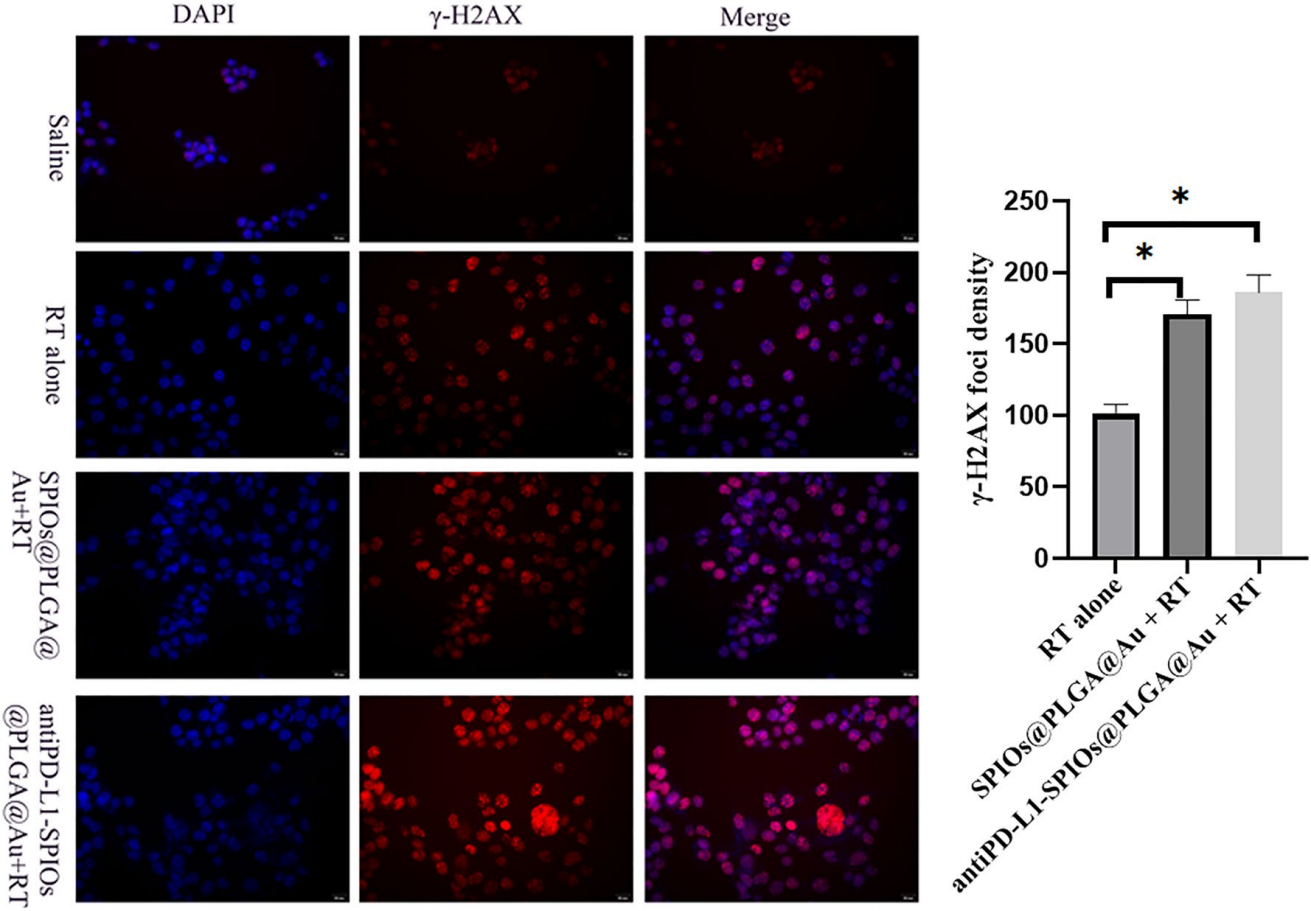
Focal fluorescence and quantitative analysis of γ-H2AX in different groups (*P < 0.05).

Under fluorescence microscope, the number of foci of γ -H2Ax increased mildly in the radiation alone group, while in SPIOs@PLGA@Au with radiation group and antiPD-L1-SPIOs@PLGA@Au with radiation group, there were more γ -H2Ax foci. The fluorescence intensity in the antiPD-L1-SPIOs@PLGA@Au with radiation group was the strongest (Fig. 4).Counting results showed that the average focal number of γ-H2AX in the SPIOs@PLGA@Au + RT and antiPD-L1-SPIOs@PLGA@Au + RT groups was 1.68 times and 1.84 times higher than that in the control group (*P* < 0.05).

#### Re-polarization effects on macrophages

**Figure 5:**
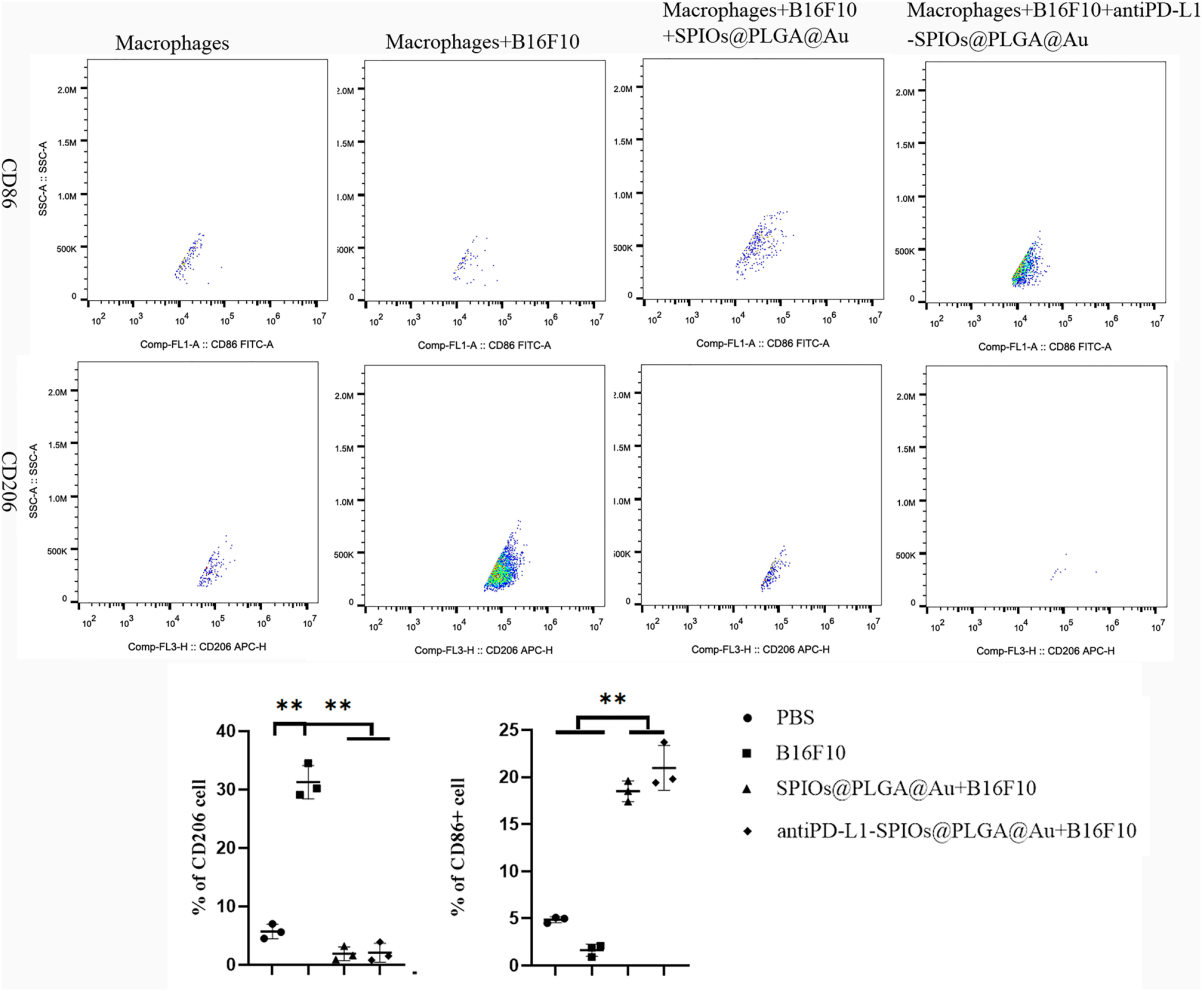
Flow cytometry of M1 and M2 macrophages after co-culture with different culture components (**P < 0.001).

It was revealed that the expression rates of CD86 and CD206 in macrophages in PBS group were similar. After co-culture with B16F10 alone, a large amount of CD206 was expressed, indicating that tumor cells can promote macrophages transform into tumor growth-promoting M2 type. After adding SPIOs@PLGA@Au or antiPD-L1-SPIOs@PLGA@Au with B16F10, M1 type macrophages expressing CD86 increased significantly, while M2 type macrophages expressing CD206 decreased significantly, indicating that nanoparticles consisting of Au and SPIOs have the function of re-polarizing TAMs (Figs. 5 and S8). However, no significant difference in the re-polarization effect between PD-L1 targeted nanoparticles and non-targeted nanoparticles (Fig. 5), indicating that the reverse effect on TAMs is mainly caused by Au-SPIOs nanoparticles, while PD-L1 has no significant effect on TMAs polarization.

Furthermore, we verified the effect of nanomaterials on TAMs polarization in the murine subcutaneous B16F10 model. The tumor bearing mice were randomly divided into four groups: saline group, radiation alone group, SPIOs@PLGA@Au with radiation group and antiPD-L1-SPIOs@PLGA@Au with radiation group. One week after different treatments, the expressions of CD206, CD86 and F4/80 proteins in the tumor tissue were detected by immunofluorescence staining. The results showed that F4/80 expression, representing the total number of macrophages, had no significant difference among the four groups. However, compared with the saline group and radiation alone group, the expression of CD86 increased significantly in the SPIOs@PLGA@Au and antiPD-L1-SPIOs@PLGA@Au groups, while the expression of CD206 decreased (Fig. S9). Moreover, targeted nanomaterials had a stronger ability to transform TAMs into M1 than the non-targeted group. The result was different from that in vitro experiment, which may be explained by that targeted nanomaterials enable more Au-SPIOs nanomaterials to actively target into tumor tissues and obtain a higher concentration, thus achieving a better re-polarizaiton effect.

### The expression of calreticulin (CRT)

As shown by immunofluorescence stain, both antiPD-L1-SPIOs@PLGA@Au and SPIOs@PLGA@Au combined with RT resulted in a higher expression of CRT compared to RT alone. The highest expression of CRT was observed in antiPD-L1-SPIOs@PLGA@Au group (Fig. S10).

### *Infiltration of CD4*^+^*and CD8*^+^*T cell in tumor*

**Figure 6:**
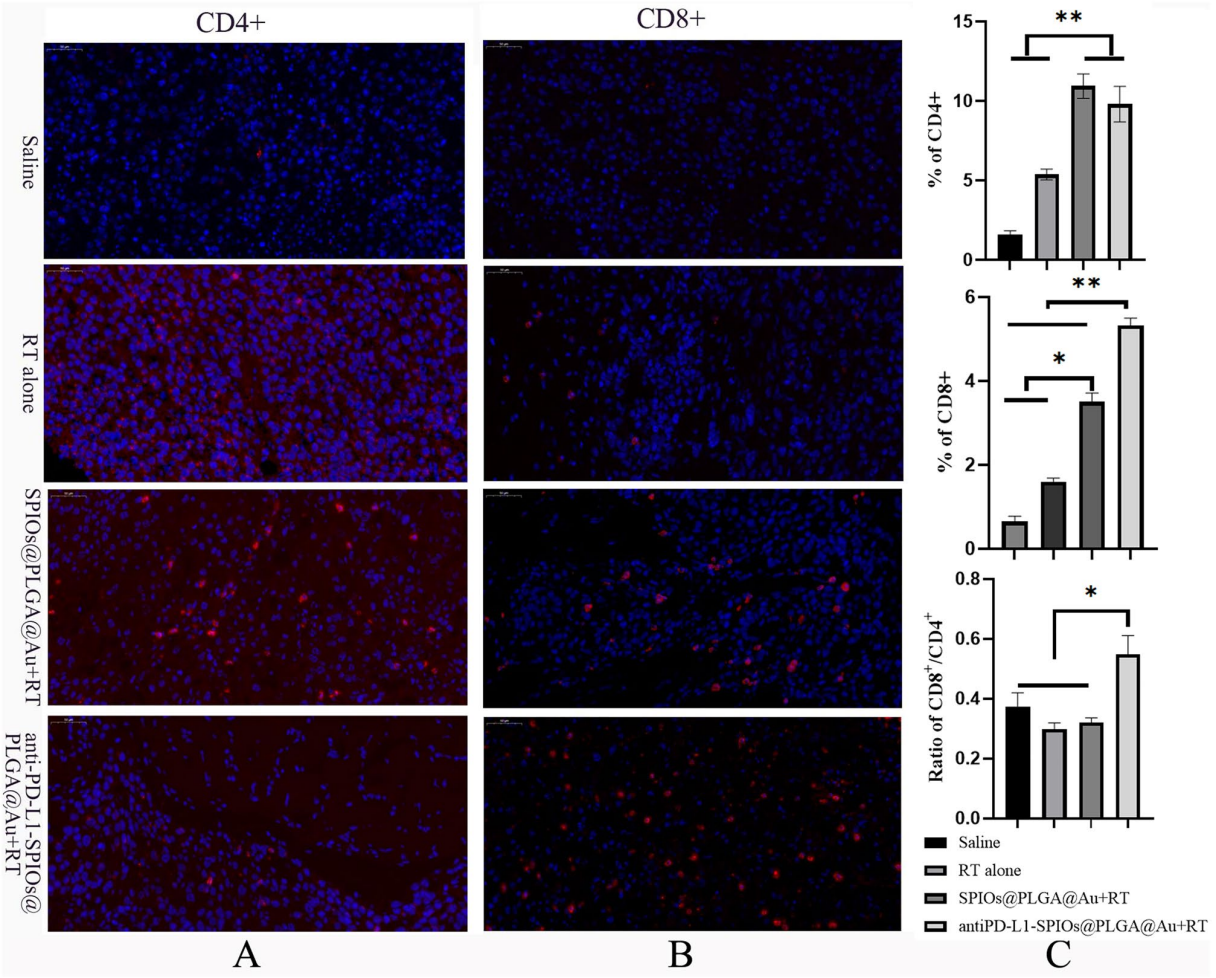
(**A**) Representative immunofluorescence images of tumor slices stained by anti-CD4 antibody (red). (**B**) Representative immunofluorescence images of tumor slices stained by anti-CD8 antibody (red). (**C**) Proportions of tumor-infiltrating CD8 + T cells and CD4 + T cells, and the ratio of CD8^+^/CD4^+^ T cells in the tumor.

In the saline group, no obvious infiltration of CD4^+^ and CD8^+^ T cell in the tumor tissues was observed. Both radiation alone and radiation combined with SPIOs@PLGA@Au increased the level of CD4^+^ and CD8^+^ T cell. The highest level of CD8 and the ratio of CD8/CD4 were achieved in radiation combined with antiPD-L1-SPIOs@PLGA@Au group, suggesting more infiltration of CD8^+^T cell (Fig. 6).

### The activating of anti-tumor immune response

Moreover, to better understand how antiPD-L1-SPIOs@PLGA@Au with radiation interacted with immunological system, we harvested tumor draining lymph nodes for analyzing the proliferation of T cell. The sera of mice one week after different treatments was collected to analyze IFN-γ, TNF-α and IL-12 level by ELISA. CD8^+^ T cell significantly increased in the presence of antiPD-L1-SPIOs@PLGA@Au compared with SPIOs@PLGA@Au, and the ratio of CD8/CD4 was higher in antiPD-L1-SPIOs@PLGA@Au group than that in the SPIOs@PLGA@Au group (Fig. S11).

In control group, Il-12 decreased one week after injection of saline, while IFN-γ and TNF-α did not change significantly. IFN-γ and TNF-α slightly increased in the radiation alone group, but there was no statistical significance. IFN-γ and IL-12 in the untargeted nanoparticles group showed a statistically significant increase. IFN-γ, TNF-α and IL-12 significantly increased in the antiPD-L1-SPIOs@PLGA@Au group, indicating that the targeted nanomaterials had a significant activation effect on the immune response (Fig. S12).

## Discussion

Radiation therapy is employed extensively for treatment of almost all types of solid tumors. Unfortunately, ionizing radiation does not discriminate between cancerous and normal cells. Thus, normal tissue damage is still the dose limiting factor that diminishes tumor cells eradication in radiation therapy. Application of tumor-specific nanoparticles in radiation therapy has aimed to improve radiotherapy effect by inducing more toxicity for tumors and less for normal tissue. On the other hand, the therapeutic effect of RT is in part dependent on an intact immune system. Radiation leads to an increased release of tumor antigens, cytokines and chemokines which promotes tumor specific T cell trafficking and priming. Unfortunately, like a double-edged sword, radiation can also create an immunosuppressive environment. Radiation resulted in upregulation of PD-L1 expression, which exhausts the number of T cells and impairs antitumor immunity^11^. To address the aforementioned obstacle, we fabricated PD-L1-targeted multifunctional nanoplatform, in which Au was coated on the surface of PLGA encapsulated with SPIOs to enhance radiosensitization of the melanoma and switch immunosuppressive microenvironment. With the addition of antiPD-L1-SPIOS@PLGA@Au, the survival of melanoma-bearing mice was significantly prolonged. All the mice survived to 20 days after the combination therapy of antiPD-L1-SPIOs@PLGA@Au and radiation. These results indicated that the combination of antiPD-L1-SPIOs@PLGA@Au and radiation strategy would be a promising antitumor approach.

Due to containing high Z-material Au with the property of high absorption and deposition of energy, antiPD-L1-SPIOs@PLGA@Au resulted in a SER of 1.4, which is comparable to that achieved by other Au formulations reported in the literature. Radiosensitivity enhancement effect can be increased with specific targeting to overexpressed receptor on the surface of tumor cell which can promoted the cellular uptake. A significantly higher radiosensitivity enhancement effect was achieved with anti-PD-L1-conjugated (SER = 1.4) versus non-conjugated nanoparticle (SER = 1.26), which could be attributed to higher cellular uptake of the targeted nanoparticles by B16F10 cells that express high level PD-L1.

Relatively larger size of the nanoparticles is helpful for longer circulation within the blood stream^12^. Targeted nanocapsules used in this study were designed to have average diameter of about 280 nm, which can have low renal clearance, reduce the non-specific uptake by normal organ and prolong the circulation time. When they reached the tumor with acid microenvironment and was irradiated by X ray, Au nanoshell would break into about 10–100 nm Au bris, which are favorable for cellular uptake^13,14^. Higher cellular uptake would result in higher radiosensitivity. AntiPD-L1-SPIOs@PLGA@Au combined with RT in this study alleviated the dilemma between the circulation time and the cellular uptake.

Shell structure with cavity inside provides an ideal platform for multi-functional applications due to their unique morphological and optical property. The nanocapsule with Au shell not only enables radiosensitization, but the shell thickness can also be fine-tuned for photothermal ablation using near infrared ray (NIR). As well, the hollow cavity can be utilized to encapsulate therapeutic agents and image contrast. Park et al. evaluated the therapeutic of DOX-loaded hollow AuNPs for combining chemo-, radio- and thermal therapy^15^. The triple combination group was shown to result in a 4.3-fold increase in tumor growth delay, as well as a 6.8-fold reduction in tumor weight. In this study we encapsulated SPIOs for highly sensitive MR imaging. The tumor accumulation of antiPD-L1-SPIOS@PLGA@Au generated a hypointensive signal in MRI, addressing low sensitivity of Au as a CT contrast agent, which requires delivery of significantly higher concentrations of Au to the tumor to generate sufficient contrast enhancement in CT (mM range) relative to the amount necessary for radiosensitization (μM range). Besides contrast for MRI, SPIOs have magnetic property. Magnetic core of the nanocomplex allowed magnetic navigation to improve tumor targeting and minimize off-target effects^16^.

Immune checkpoint inhibitors (ICIs) targeting PD-1, PD-L1 and CTLA-4 can relieve the restraint of antitumor T cell immunity and improve the prognosis of patients with advanced cancer^17^. However, the curative effect of ICIs depends on the T cells activation. Immunosuppressive cells contributing to the immune evasion will lead to the failure of ICIs^18^. Therefore, how to inhibit the activity of these immunosuppressive cells is currently a matter of concern, among which TAM is one of the typical immunosuppressive cells that occupies a significant part of tumor mass^19–21^. TAMs were able to inhibit the mature of antigen present cells (APCs) and CD8 + T cell-mediated antitumor immune responses by producing high level of IL-10^22,23^. Recently, SPIOs were found to be able modulate immune microenvironment. It was shown that SPIOs polarized tumor-associated macrophages from M2- to M1 phenotype, which released reactive oxygen species to induce tumor cell killing^5,24^. In this study, treating with SPIOs-containing nanoparticle significantly elevated the expression of CD86 and reduced the expression of CD206 compared to RT alone, indicating the polarization to M1.

Moreover, it’s demonstrated that macrophages are the predominant immune cells that express PD-L1^25,26^. These PD-L1^+^ TAMs could mediate CD8^+^ T cell dysfunction via the PD-1/PD-L1 interaction. TAMs have been regarded as carriers of checkpoint ligands that are upregulated in response to TME-derived factors, resulting in immune exhaustion via the checkpoint ligand/receptor interaction in a cell-to-cell contact manner. Therefore, the blocking effect of ICIs on the checkpoint molecules expressed on TAMs is increasingly attracting attention^27^. AntiPD-L1-SPIOs@PLGA@Au realized dual-targeting strategy, achieving the combination of reprogramming and elimination of TAM and inhibition of immune checkpoint. AntiPD-L1-SPIOs@PLGA@Au with local RT not only increased the number of CD8^+^ cells in tumor but also enhanced the proliferation of CD8^+^ cells in the tumor draining lymph nodes (TDLN) and elevated the level of serum IFN, indicating the activation of systemic antitumor immunity. A further understanding of their intracellular regulatory mechanisms will be helpful for precise application of TAMs targeted therapy and ICI treatment.

## Conclusions

We successfully prepared antiPD-L1-SPIOs@PLGA@Au nanoparticles, which realized the combination of radiotherapy and immunotherapy and enhanced the antitumor effect. With the application of antiPD-L1-SPIOs@PLGA@Au, we enabled to target PD-L1, increase the accumulated concentration of nanoparticle in tumor and enhance B16F10 sensitivity to radiation. By inducing the repolarization from M2 type to M1 and elevating the expression of CRT, antiPD-L1-SPIOs@PLGA@Au was able to switch the immunosuppressive environment. Combined with the effect of blocking the PD-1/PD-L1 checkpoint pathway, antiPD-L1-SPIOs@PLGA@Au and RT activated the tumor specific immunity ultimately. These results indicated that the combination of antiPD-L1-SPIOS@PLGA@Au and RT strategy would be a promising approach to effective antitumor therapy.

## Supplementary Information

Supplementary Information.

## Data Availability

Requests for materials should be addressed to HY.

## References

[1] Delaney G, Jacob S, Featherstone C, Barton M (2005). The role of radiotherapy in cancer treatment: Estimating optimal utilization from a review of evidence-based clinical guidelines. Cancer.

[2] Her S, Jaffray DA, Allen C (2017). Gold nanoparticles for applications in cancer radiotherapy: Mechanisms and recent advancements. Adv. Drug Deliv. Rev..

[3] Bulte JW, Kraitchman DL (2004). Iron oxide MR contrast agents for molecular and cellular imaging. NMR Biomed..

[4] McQuade C, Al Zaki A, Desai Y (2015). A multifunctional nanoplatform for imaging, radiotherapy, and the prediction of therapeutic response. Small.

[5] Zanganeh S, Hutter G, Spitler R (2016). Iron oxide nanoparticles inhibit tumour growth by inducing pro-inflammatory macrophage polarization in tumour tissues. Nat. Nanotechnol..

[6] Wang Y, Wang H, Yao H, Li C, Fang JY, Xu J (2018). Regulation of PD-L1: Emerging routes for targeting tumor immune evasion. Front. Pharmacol..

[7] Qu QX, Xie F, Huang Q, Zhang XG (2017). Membranous and cytoplasmic expression of PD-L1 in ovarian cancer cells. Cell Physiol. Biochem..

[8] Topalian SL, Drake CG, Pardoll DM (2015). Immune checkpoint blockade: a common denominator approach to cancer therapy. Cancer Cell.

[9] Karam SD, Raben D (2019). Radioimmunotherapy for the treatment of head and neck cancer. Lancet Oncol..

[10] Zheng D, Wan C, Yang H (2020). Her2-targeted multifunctional nano-theranostic platform mediates tumor microenvironment remodeling and immune activation for breast cancer treatment. Int. J. Nanomed..

[11] Kikuchi M, Clump DA, Srivastava RM (2017). Preclinical immunoPET/CT imaging using Zr-89-labeled anti-PD-L1 monoclonal antibody for assessing radiation-induced PD-L1 upregulation in head and neck cancer and melanoma. Oncoimmunology.

[12] Dreaden EC, Austin LA, Mackey MA, El-Sayed MA (2012). Size matters: Gold nanoparticles in targeted cancer drug delivery. Ther. Deliv..

[13] Huo S, Ma H, Huang K (2013). Superior penetration and retention behavior of 50 nm gold nanoparticles in tumors. Cancer Res..

[14] Adair JH, Parette MP, Altinoglu EI, Kester M (2010). Nanoparticulate alternatives for drug delivery. ACS Nano.

[15] Park J, Park J, Ju EJ (2015). Multifunctional hollow gold nanoparticles designed for triple combination therapy and CT imaging. J. Control Release.

[16] Chiang CS, Lin YJ, Lee R (2018). Combination of fucoidan-based magnetic nanoparticles and immunomodulators enhances tumour-localized immunotherapy. Nat. Nanotechnol..

[17] Topalian SL, Hodi FS, Brahmer JR (2012). Safety, activity, and immune correlates of anti-PD-1 antibody in cancer. N. Engl. J. Med..

[18] Tumeh PC, Harview CL, Yearley JH (2014). PD-1 blockade induces responses by inhibiting adaptive immune resistance. Nature.

[19] Yang Q, Guo N, Zhou Y, Chen J, Wei Q, Han M (2020). The role of tumor-associated macrophages (TAMs) in tumor progression and relevant advance in targeted therapy. Acta Pharm. Sin. B..

[20] Meng Y, Beckett MA, Liang H (2010). Blockade of tumor necrosis factor alpha signaling in tumor-associated macrophages as a radiosensitizing strategy. Cancer Res..

[21] Pollard JW (2004). Tumour-educated macrophages promote tumour progression and metastasis. Nat. Rev. Cancer.

[22] Kitamura T, Qian BZ, Pollard JW (2015). Immune cell promotion of metastasis. Nat. Rev. Immunol..

[23] Akalu YT, Rothlin CV, Ghosh S (2017). TAM receptor tyrosine kinases as emerging targets of innate immune checkpoint blockade for cancer therapy. Immunol. Rev..

[24] Li K, Lu L, Xue C (2020). Polarization of tumor-associated macrophage phenotype via porous hollow iron nanoparticles for tumor immunotherapy in vivo. Nanoscale.

[25] Wu K, Kryczek I, Chen L, Zou W, Welling TH (2009). Kupffer cell suppression of CD8+ T cells in human hepatocellular carcinoma is mediated by B7–H1/programmed death-1 interactions. Cancer Res..

[26] Kuang DM, Zhao Q, Peng C (2009). Activated monocytes in peritumoral stroma of hepatocellular carcinoma foster immune privilege and disease progression through PD-L1. J. Exp. Med..

[27] Gordon SR, Maute RL, Dulken BW (2017). PD-1 expression by tumour-associated macrophages inhibits phagocytosis and tumour immunity. Nature.

