# Supplementary material for: AntiPD-L1 antibody conjugated Au-SPIOs nanoplatform for enhancing radiosensitivity and triggering anti-tumor immune response
Source: Sci Rep. 2022 Nov 15;12:19542. doi: 10.1038/s41598-022-23434-z (PMC9666506; doi:10.1038/s41598-022-23434-z)
Supplement: Supplementary file 1 — Supplementary Information. [file 41598_2022_23434_MOESM1_ESM.docx]

Supplementary materials

Antibodies used:

Anti-PD-L1 (Clone: 10F.9G2, Catalog: BE0101, BioXcell)

Anti-PD-L1: PE/Cyanine7 (Clone: 10F.9G2, Catalog: 124314, Biolegend)

Anti-PD-L1: FITC (Clone: 28-8, Catalog: ab224027, Abcam)

Anti-CD3: PE (Clone: 17A2, Catalog: 100206, Biolegend)

Anti-CD4: APC (Clone: RM4-5, Catalog: 100516, Biolegend)

Anti-CD8: FITC (Clone: 53-6.7, Catalog: 100706, Biolegend)

Anti-CD11b: PE/CY5 (Clone: M1/70, Catalog: 101209, Biolegend)

Anti-F4/80: PE (Clone: BM8 Catalog: 123109, Biolegend)

Anti-CD206: APC (Clone: C068C2 Catalog: 141707, Biolegend)

Anti-CD86: FITC (Clone: GL-1 Catalog: 105005, Biolegend)

Supplementary figures


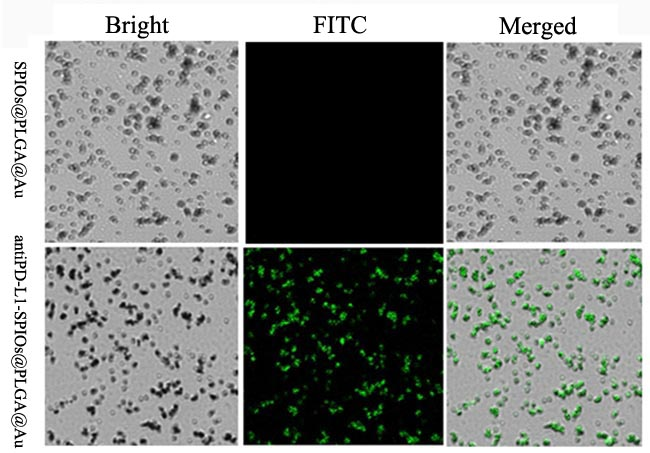


Figure S1. Confocal laser scanning microscopy images of SPIOs@PLGA@Au and antiPD-L1-SPIOs@PLGA@Au labelled with FITC.

Figure S2. The effect of radiation on the morphology of antiPD-L1-SPIOs@PLGA@Au. (A) AntiPD-L1-SPIOs@PLGA@Au stayed stable for 7 days with unchanged diameter. The change of morphology of antiPD-L1-SPIOs@PLGA@Au after radiation (B: before radiation; C: after radiation).


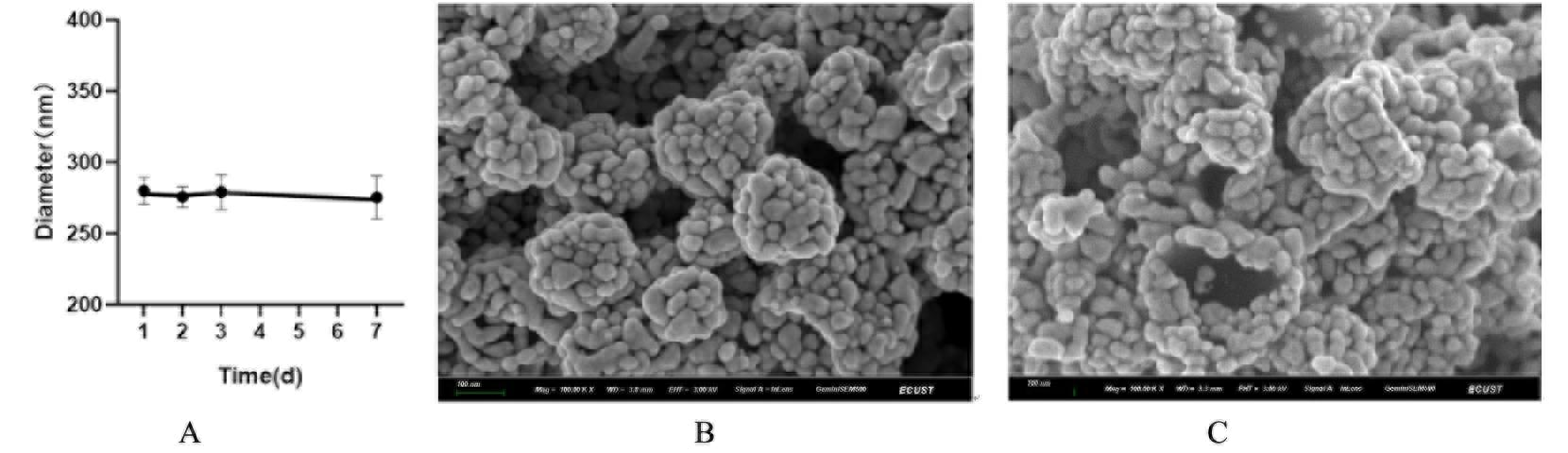

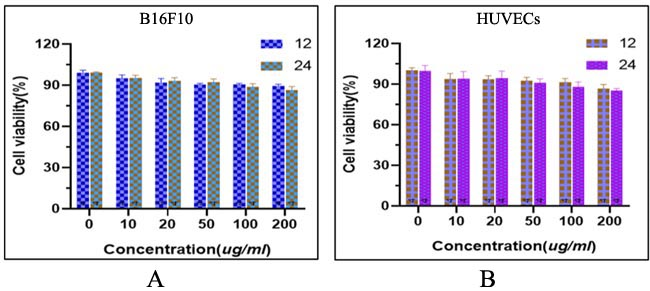


Figure S3. Cell viability of (A) B16F10 cell and (B) HUVECs after incubation (12h and 24h) with various concentrations of antiPD-L1-SPIO@PLGA@Au.





Figure S4. (A) The blood biochemistry of mice treated with antiPD-L1-SPIOs@PLGA@Au or saline (control). (B)Body weight of mice treated with antiPD-L1-SPIOs@PLGA@Au or saline (control) (C) Microscope images of H&E stained tissues in heart, liver, spleen, lung and kidney.


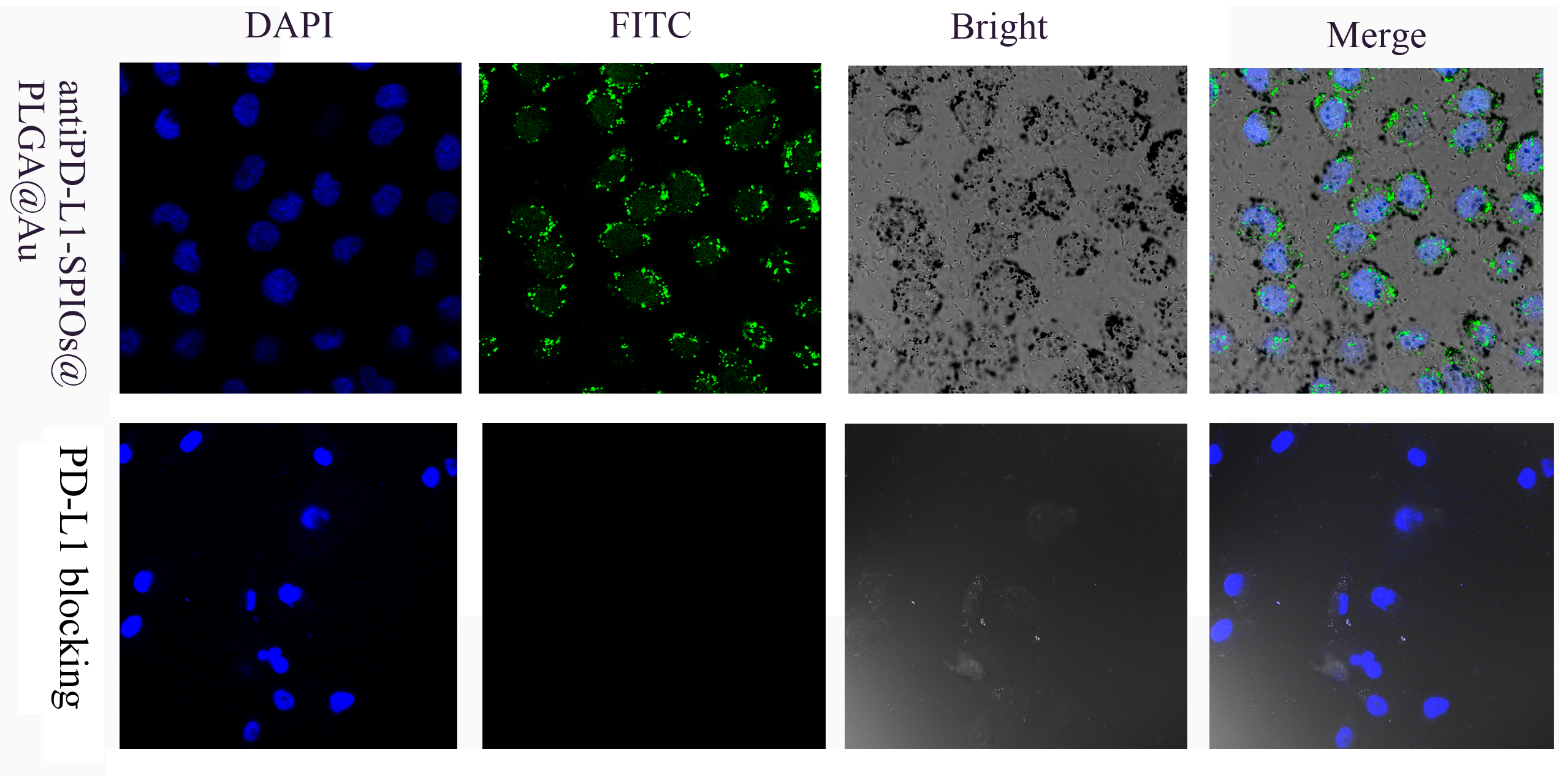


Figure S5. AntiPD-L1-SPIOs@PLGA@Au targeting to B16F10 cell, with and without antiPD-L1 antibody blocking.


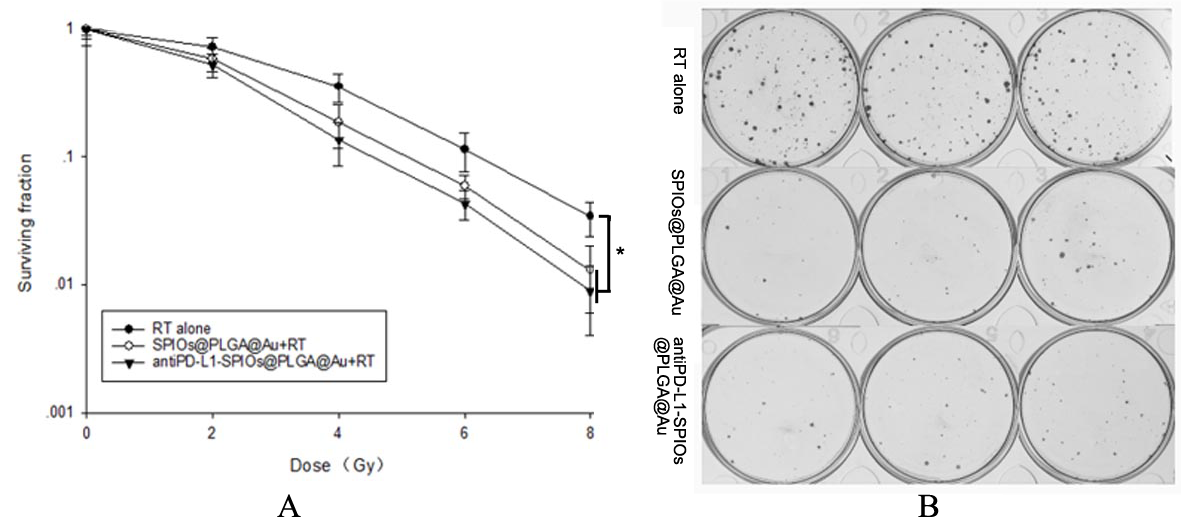


Figure S6. (A) Clonogenic assay of B16F10 cells treated with antiPD-L1-SPIOs@PLGA@Au, SPIOs@PLGA@Au or no nanoparticles and given radiation doses (*<0.05). (B) Clone formation of B16F10 cells irradiated with 4Gy in various groups.


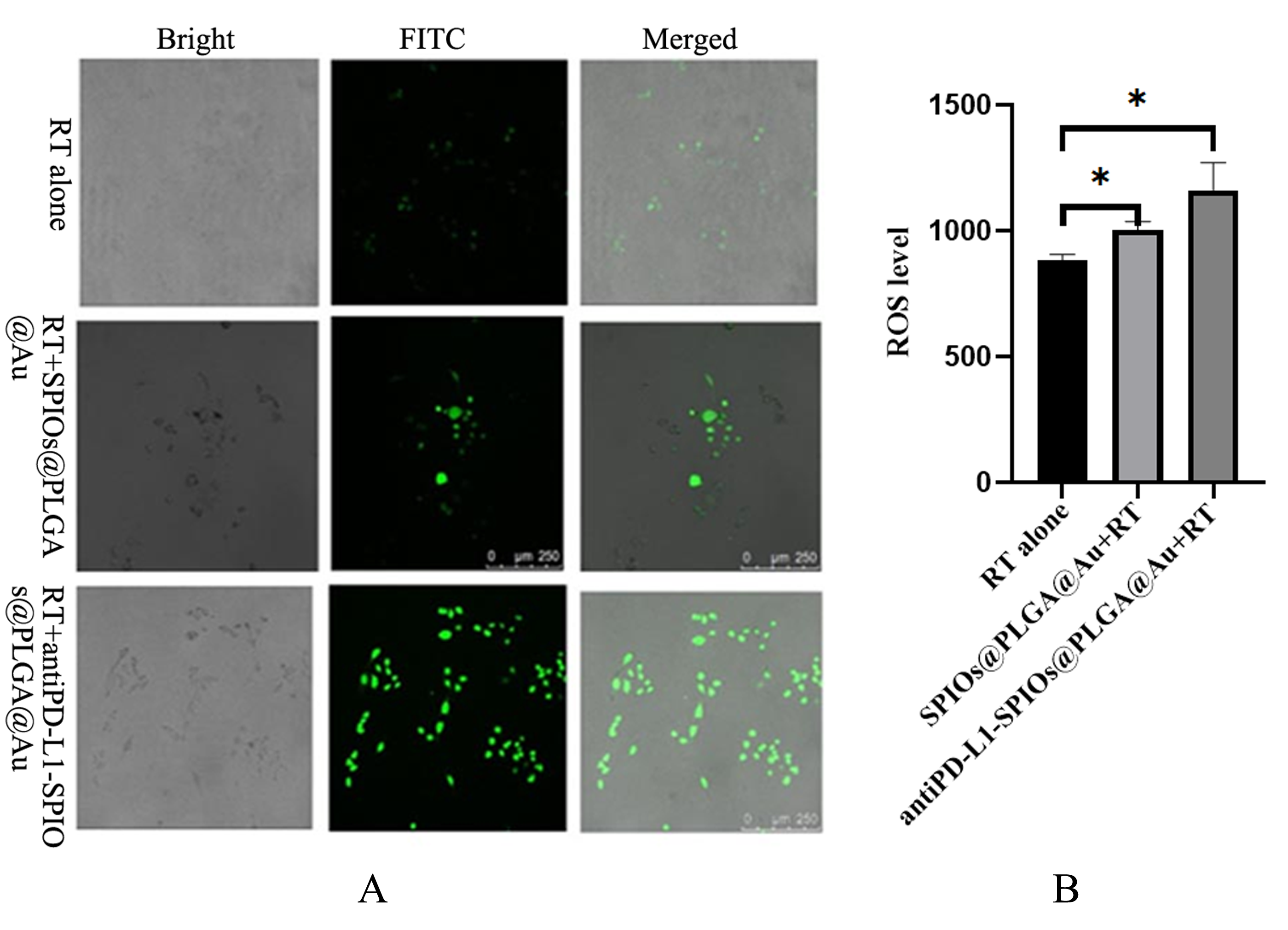


Figure S7. (A) Immunofluorescent images of ROS production induced by radiation in B16F10 cells incubated with antiPD-L1-SPIOs@PLGA@Au, SPIOs@PLGA@Au or without nanoparticles. (B) Quantitative analysis of ROS in each treatment group (*<0.05).


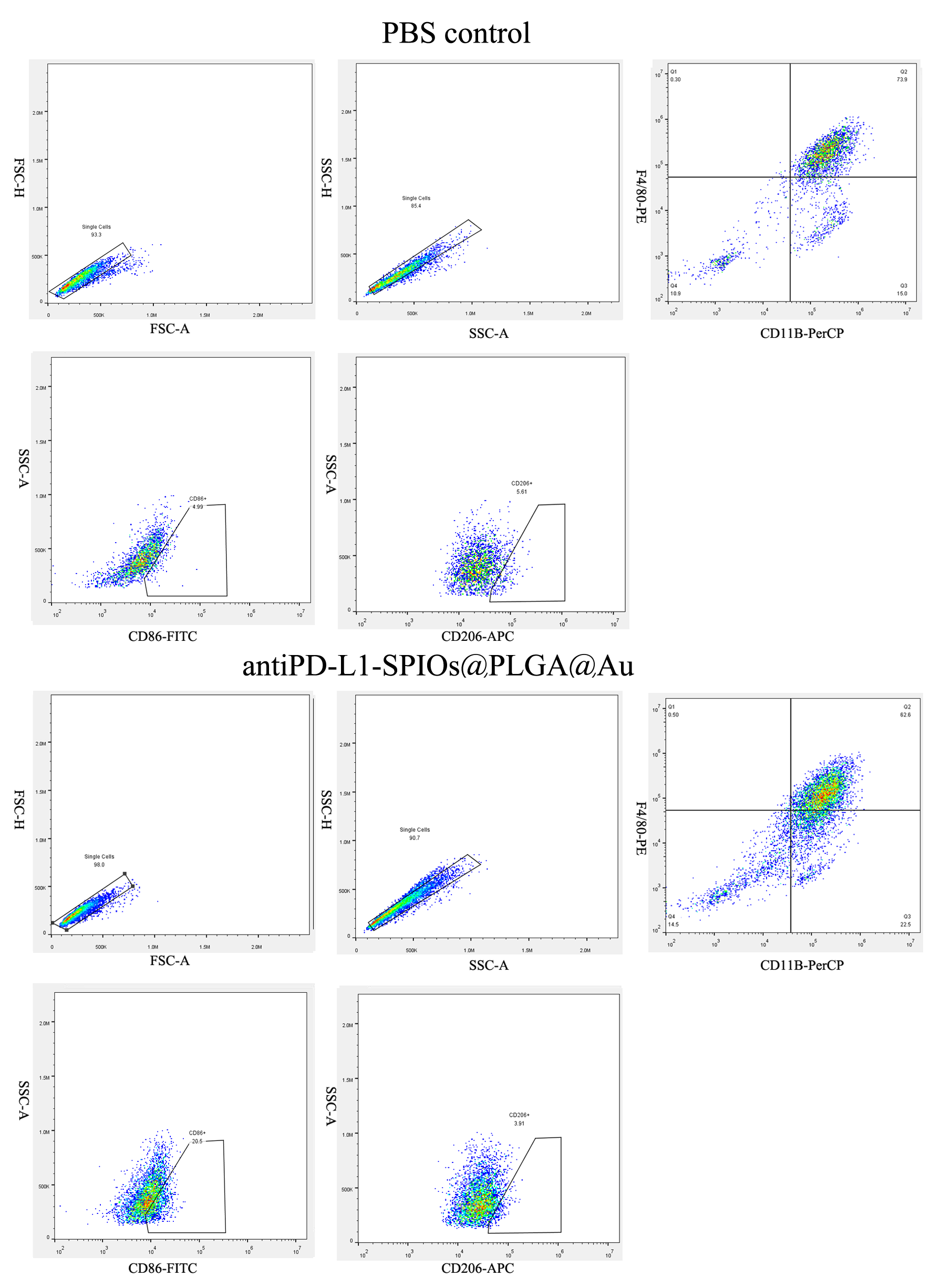


Figure S8. Flow cytometry gating strategy to assess M1 type (CD11b^+^F4/80^+^CD86^+^) and M2 type macrophage (CD11b^+^F4/80^+^CD206^+^) was performed with exclusion

of doublets by FSC and SSC, selection of CD11B^+^ and F4/80+, and further staining using CD86 and CD206 mAbs with appropriate fluorescent dyes


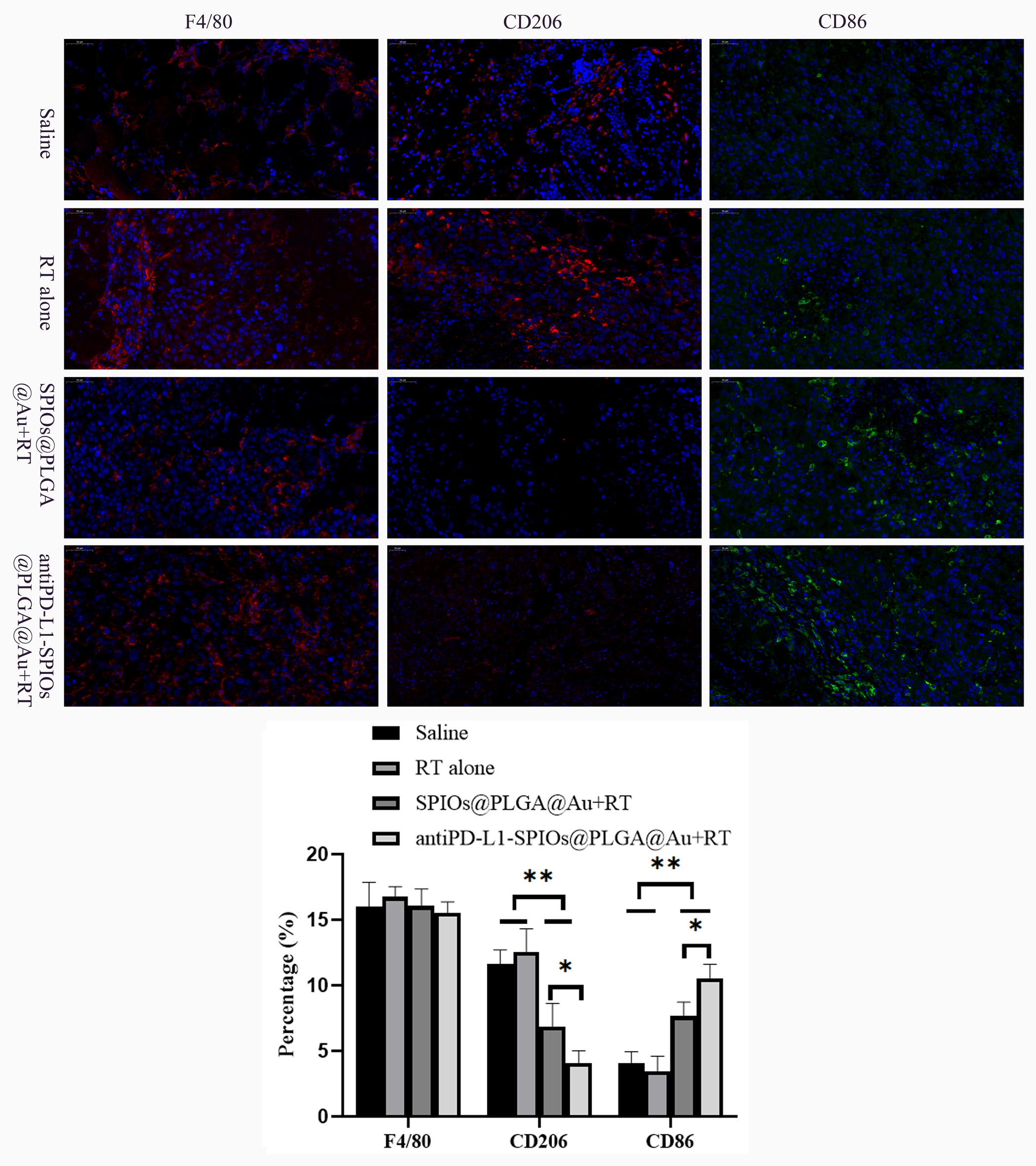


Figure S9. Immunofluorescence image and quantitative analysis of tumor sections stained with the macrophage marker of F4/80 (red), the M2 macrophage marker of CD206 (red) and the M1 macrophage marker of CD86 (green).


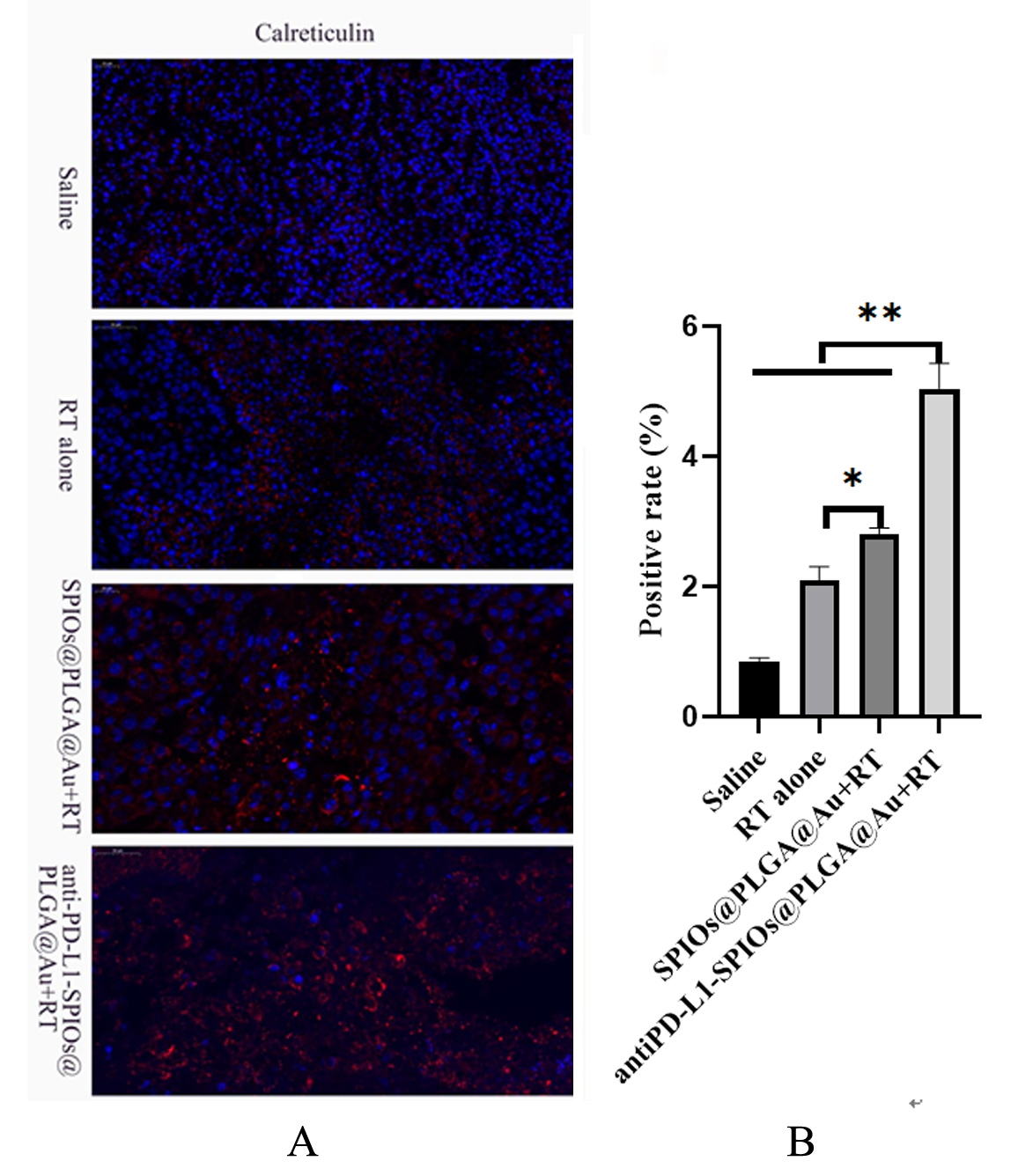


Figure S10. (A) CRT immunofluorescence staining image of tumor tissue; (B) Quantitative analysis of positive area ratio of CRT immunofluorescence staining images of tumor tissues (*<0.05; **<0.001).


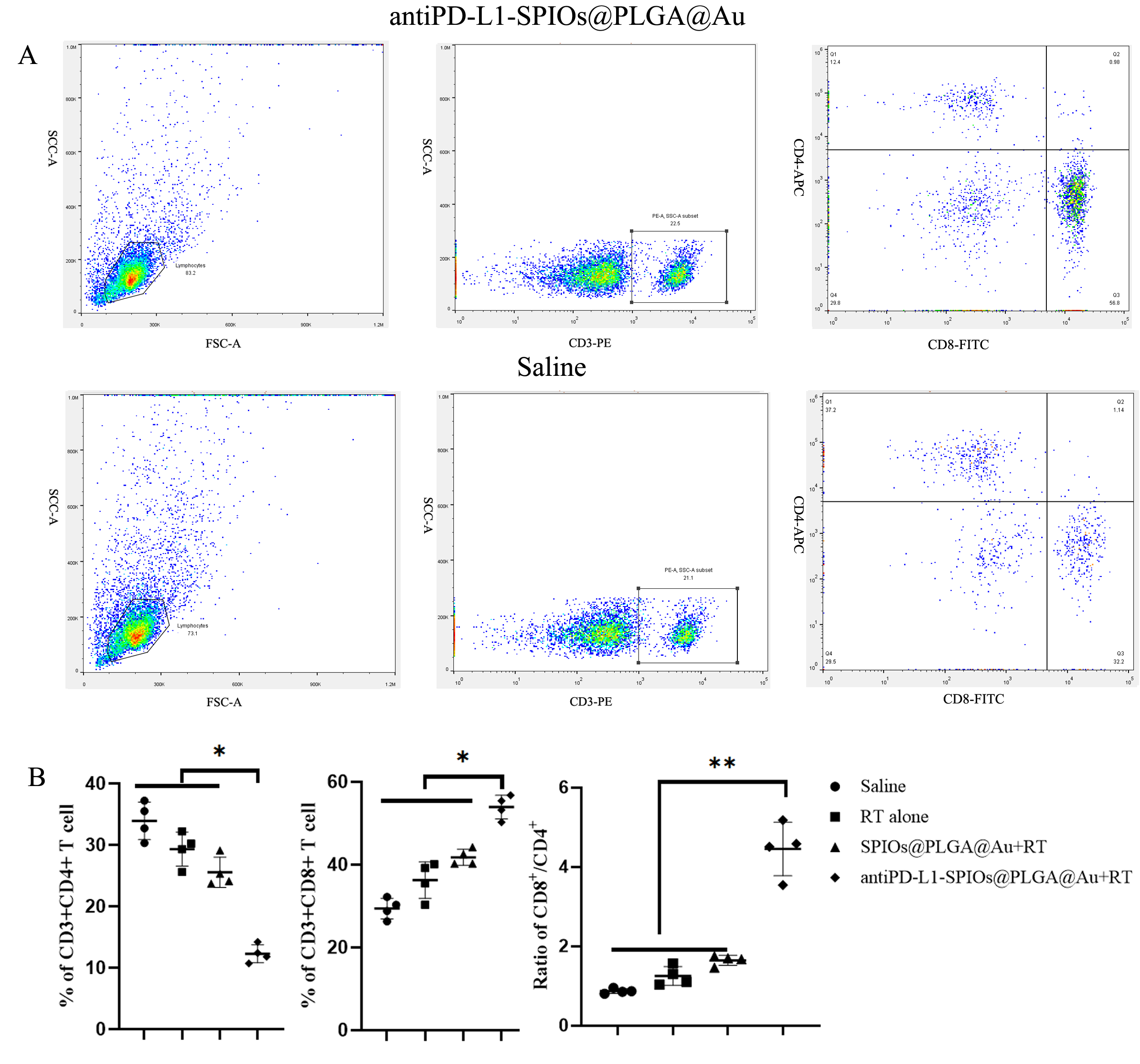


Figure S11. (A) Flow cytometry gating strategy to determine T cell lymph cells based on the size selection by FSC-A and SCC-A, selection of CD3^+^ cell, and further staining with CD8 and CD4 with appropriate fluorescent dyes to select CD3+CD8+ or CD3+CD4+ T cell. (B) Proportions of CD3^+^CD8^+^ T cells and CD3^+^CD4^+^ T cells and the ratio of CD8^+^/CD4^+^ cells in tumor draining lymph nodes (*<0.05; **<0.001).


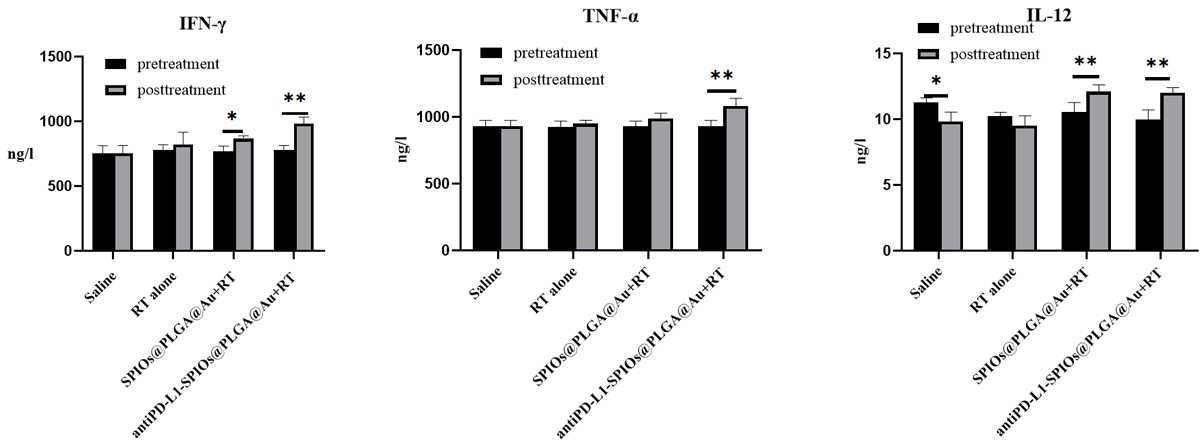


Figure S12. Cytokine levels in sera of mice in different groups.
